# Corpus Callosotomy for Atonic Drop Seizures in Bilateral Malformations of Cortical Development: A Systematic Review of Literature

**DOI:** 10.7759/cureus.81359

**Published:** 2025-03-28

**Authors:** Ethan Kosco, Warren Back, Vito Lucarelli, Aditya Acharya, Andrew Waack, Jason Schroeder

**Affiliations:** 1 Department of Neurological Surgery, The University of Toledo College of Medicine and Life Sciences, Toledo, USA

**Keywords:** atonic seizure, cortical dysplasia, drop attack, hemimegalencephaly, heterotopia, lissencephaly, malformations of cortical development, pachygyria, polymicrogyria, tuberous sclerosis

## Abstract

Malformations of cortical development (MCD) frequently manifest with epilepsy, often refractory to medical treatment. Atonic seizures, prevalent in MCD, pose significant challenges. Surgical interventions like corpus callosotomy are considered when medical control fails. However, debate persists over optimal techniques, particularly in bilateral MCD (BMCD). A systematic review following Preferred Reporting Items for Systematic Reviews and Meta-Analyses (PRISMA) guidelines analyzed studies on corpus callosotomy for atonic seizures in BMCD. Eligible studies encompassed patients undergoing anterior or total callosotomy post-failed medical therapy. Data extraction and quality assessment were performed independently by two reviewers.

Nineteen primary articles involving 187 patients were included, analyzing outcomes of total callosotomy (TC) versus partial callosotomy (PC) in various BMCD types. TC demonstrated favorable seizure cessation rates, with notable improvements in tuberous sclerosis and cortical dysplasia cases. PC showed efficacy, especially in subcortical band heterotopia. Studies highlighted the need for long-term follow-up to assess sustained efficacy and neurocognitive impacts. This review underscores the potential of total callosotomy in severe refractory epilepsy associated with BMCD while acknowledging the utility of partial callosotomy in selected cases. Individualized approaches guided by pathology and seizure phenotype are crucial. Future research should focus on optimizing surgical techniques and exploring adjunctive therapies. Corpus callosotomy offers promise in managing atonic seizures in BMCD. Tailored surgical strategies, guided by comprehensive patient assessment, are essential. Continued research is imperative to refine techniques and enhance outcomes for this challenging patient population.

## Introduction and background

Malformations of cortical development (MCD) are congenital brain disorders resulting from the interruption of normal neuroglial migration, proliferation, or cortical organization [1]. The MCD classification includes several disorders, including tuberous sclerosis, cortical dysplasia, hemimegalencephaly, lissencephaly, pachygyria, heterotopia, and polymicrogyria. Each of these MCDs has a unique pathophysiological mechanism and clinical presentation. However, epilepsy and atonic seizures are common features among MCDs [1]. These are often refractory to pharmacologic treatment and therefore require surgical intervention.

Several surgical approaches have been described for MCD with medically refractory atonic seizures, including focal resection, hemispherectomy, neuromodulation, and corpus callosotomy [1-3]. Favorable outcomes with resective surgery have been well described in MCD with unilateral localized epileptogenic foci [4]. However, resection cannot be performed in cases of bilateral MCD (BMCD) [1,4]. Instead, corpus callosotomy is an appropriate alternative to resection [1]. There remains considerable debate regarding the optimal surgical technique for corpus callosotomy in BMCD. It is presumed that more extensive anterior-posterior lesioning leads to superior seizure control at the expense of an increased risk of adverse neuropsychological effects. Variable outcomes have been reported with these different approaches. Some authors advocate for a complete callosotomy for optimal seizure control, whereas others encourage a more conservative partial (anterior) callosotomy. Neither technique has demonstrated superiority, and there is no consensus regarding the optimal extent of callosal lesioning in patients with bilateral MCDs.

We aimed to compare the rates of seizure cessation between partial callosotomy and total callosotomy in patients with BMCD associated with atonic drop attack seizures.

Methods

*Protocol and Search Strategy* 

**Figure 1:**
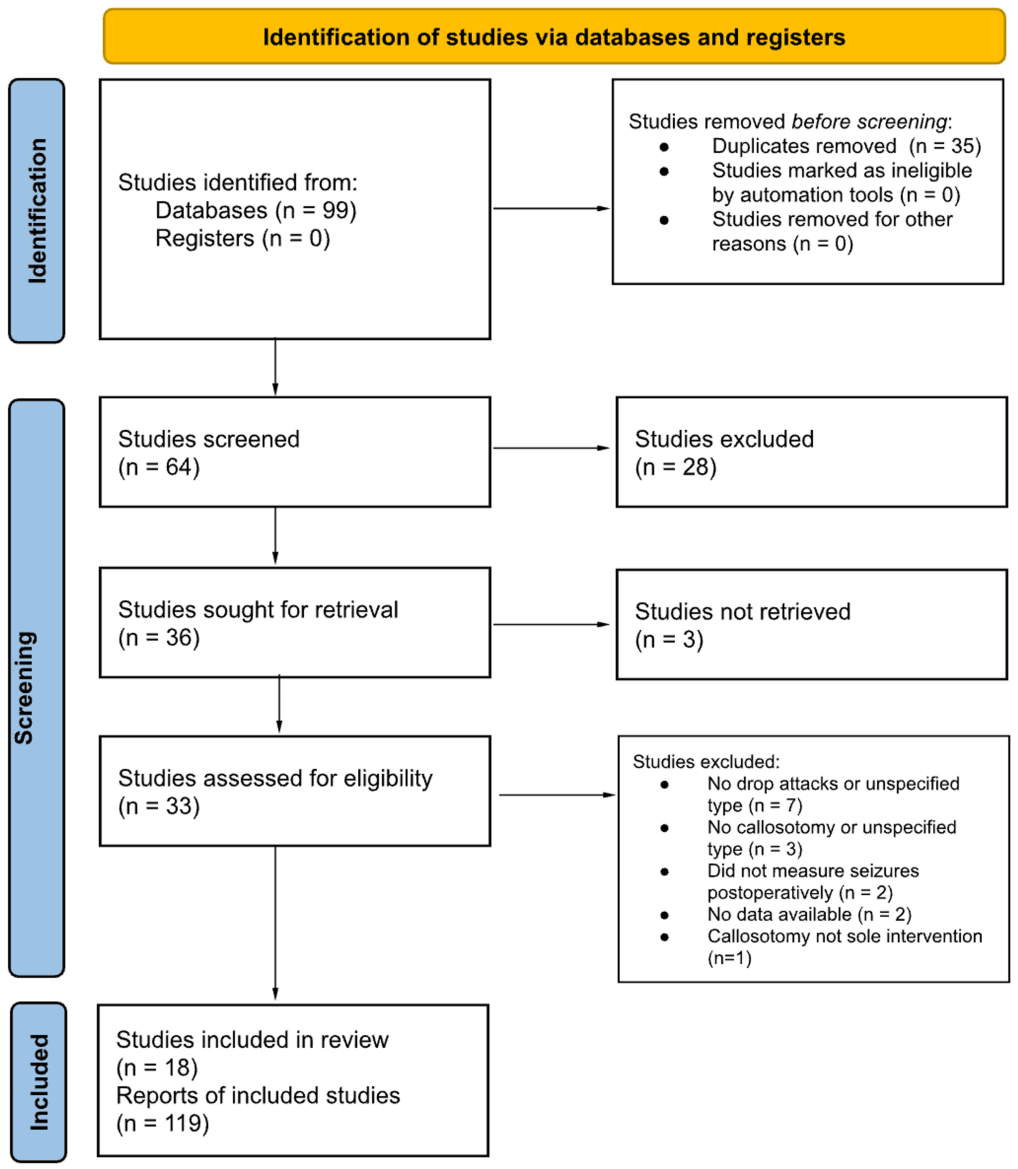
PRISMA flow diagram for study inclusion. PRISMA: Preferred Reporting Items for Systematic Reviews and Meta-Analyses

We performed a systematic search of published English literature in the PubMed, Web of Science, and Cochrane Library databases from 1990-2020 per Preferred Reporting Items for Systematic Reviews and Meta-Analyses (PRISMA) guidelines (2021) (Figure 1) [5]. The following search terms were used to identify studies of interest: atonic drop attack OR drop seizure OR akinetic seizure OR astatic seizure OR drop attack AND callosotomy AND malformation of cortical development OR tuberous sclerosis OR cortical dysplasia, lissencephaly OR pachygyria OR heterotopia OR heterotopia OR polymicrogyria. The inclusion criteria consisted of patients with BMCD and atonic drop seizures who underwent either partial or total corpus callosotomy after failing medical therapy.

Eligibility Criteria, Study Selection, and Data Extraction

Primary studies, including case reports, case series, and cohort studies, were included in our review. The inclusion criteria consisted of patients with BMCD and atonic drop seizures who underwent either partial or total corpus callosotomy after failing medical therapy. BMCD types included tuberous sclerosis, cortical dysplasia, lissencephaly, pachygyria, heterotopia, and polymicrogyria. Letters to the editors, review articles, and studies that did not specify the type of callosotomy or type of seizure were excluded from the analysis. Secondary review articles were not included to prevent inadvertently recording the same individual case multiple times. To compare the efficacy between TC and PC, surgical outcomes were defined as either “freedom from drop attacks” or “decrease drop attacks.” For a paper to be defined as successfully decreasing drop attacks, it had to report data describing patients who received the procedure and had a statistically significant decrease in drop attacks.

Data extraction and manuscript review were carried out independently by two reviewers (EK and VL), which initially yielded 95 studies. The total collection of gathered manuscripts was manually screened for duplicates, and 31 studies were subsequently removed. Following duplicate removal, manuscripts were screened by title and abstract, and 28 studies were excluded. A complete full-text review was then conducted to determine study eligibility, and 15 studies did not meet eligibility criteria at this stage. Lastly, three studies were inaccessible to the reviewers and were thus removed. Disagreement regarding study inclusion at any stage of review was resolved by a third reviewer (AW).

Relevant case information from eligible studies was extracted and recorded in a standardized Microsoft Excel (Redmond, WA: Microsoft Corp.) file. Extracted information included are as follows: (1) patient demographics (age, sex, comorbidities), (2) cortical pathology (MCD type), (3) epilepsy features, (4) antiseizure medications (ASM) protocol, (5) callosotomy (partial or total), (6) surgical outcome, and (7) miscellaneous information.

Risk of Bias

Case reports and case series are inherently biased and are not typically included in systematic reviews and meta-analyses. However, standardized evaluation criteria have been developed for assessing the bias of included case reports [6]. Both reviewers (EK and VL) assessed the quality of included case reports and case series with a standardized tool created by Murad et al. and the Joanna Briggs Institute “Checklist for Case Reports” guidelines [6]. Reports were rated as low, moderate, or high risk of bias.

Results

A total of 18 primary review articles analyzing 119 patients were included in the study. Of these, nine focused on total callosotomy (TC), six on partial callosotomy (PC), and three evaluated both, either by comparison or patients undergoing both procedures. The study distribution of BMCD diagnosis was four on tuberous sclerosis (three TC, one both), five on cortical dysplasia (two TC, three PC), five on heterotopia (one TC, three PC, one both), one on lissencephaly (TC), and one on polymicrogyria (TC). Although several studies assessed both TC and PC, only one patient received a partial callosotomy followed by a total callosotomy due to the persistence of symptoms. Table 1 shows the distribution of patients by diagnosis and procedure type. Tables 2-6 show the outcomes of the bilateral malformation of cortical development (BMCD) subtype. Table 7 demonstrates overall outcomes without regard to the BMCD subtype.

**Table 1:** Procedure distribution by BMCD subtype. *Total number of patients with drop attacks that underwent callosotomy procedure. TC: total callosotomy; PC: partial callosotomy; BMCD: bilateral malformations of cortical development

Variables	Patients* (187)	TC (117)	PC (69)	Both (1)
Tuberous sclerosis	42 (22.5%)	40 (34.2%)	2 (2.9%)	0
Cortical dysplasia	60 (32.1%)	4 (3.4%)	56 (81.2%)	0
Heterotopia	11 (5.9%)	5 (4.3%)	5 (7.2%)	1 (100%)
Lissencephaly	16 (8.6%)	16 (13.7%)	0	0
Polymicrogyria	1 (0.5%)	1 (0.9%)	0	0

**Table 2:** Tuberous sclerosis patient outcomes. DA: drop attacks; FDA: freedom from drop attacks; TC: total callosotomy; PC: partial callosotomy

Tuberous sclerosis	Total (42)	TC (40)	PC (2)	Both (0)
FDA	23 (54.8%)	21 (52.5%)	2 (100%)	-
Decrease DA	1 (2.4%)	1 (2.5%)	0	-
No change	18 (42.9%)	18 (45%)	0	-

**Table 3:** Cortical dysplasia patient outcomes. *Results from related studies only specified a decrease in seizure frequency, not FDA. DA: drop attacks; FDA: freedom from drop attacks; TC: total callosotomy; PC: partial callosotomy

Cortical dysplasia	Total (60)	TC (4)	PC (56)	Both (0)
FDA	4 (6.7%)	2 (50%)	2 (3.6%)	-
Decrease DA	41 (68.3%)	1 (25%)	40* (71.4%)	-
No change	15 (25%)	1 (25%)	14 (25%)	-

**Table 4:** Heterotopia patient outcomes. *This patient received PC with no improvement and then subsequently underwent TC with FDA afterwards. DA: drop attacks; FDA: freedom from drop attacks; TC: total callosotomy; PC: partial callosotomy

Heterotopia	Total (11)	TC (5)	PC (5)	Both (1)
FDA	3 (27.3%)	0	2 (40%)	1* (100%)
Decrease DA	4 (36.4%)	3 (60%)	1 (20%)	0
No change	4 (36.4%)	2 (40%)	2 (40%)	0

**Table 5:** Lissencephaly patient outcomes. DA: drop attacks; FDA: freedom from drop attacks; TC: total callosotomy; PC: partial callosotomy

Lissencephaly	Total (16)	TC (16)	PC (0)	Both (0)
FDA	16 (100%)	16 (100%)	-	-
Decrease DA	0	0	-	-
No change	0	0	-	-

**Table 6:** Polymicrogyria patient outcomes. DA: drop attacks; FDA: freedom from drop attacks; TC: total callosotomy; PC: partial callosotomy

Polymicrogyria	Total (1)	TC (1)	PC (0)	Both (0)
FDA	1 (100%)	1 (100%)	-	-
Decrease DA	0	0	-	-
No change	0	0	-	-

**Table 7:** Overall patient outcomes. DA: drop attacks; FDA: freedom from drop attacks; TC: total callosotomy; PC: partial callosotomy

All patients	Total (187)	TC (117)	PC (69)	Both (1)
FDA	98 (52.4%)	87 (74.3%)	10 (14.5%)	1 (100%)
Decrease DA	46 (24.6%)	5 (4.3%)	41 (59.4%)	0
No change	43 (23.0%)	25 (21.4%)	18 (26.1%)	0

Patients with BMCD who received a partial callosotomy (PC) reported overall fewer drop attacks. PC remains the most widely used surgical management option for BMCD, with 53% of the cases in this report utilizing the procedure. However, different BMCDs often favored specific surgical approaches.

In this study, 29 out of 31 tuberous sclerosis patients received TC, with outcomes as follows: 15 experienced freedom from drop attacks (FDA), one experienced a decrease in drop attacks (DDA), and 13 reported no change (NC). Two tuberous sclerosis patients underwent a partial callosotomy (PC), both reporting fewer drop attacks (Table 2). For cortical dysplasia, 60 patients were treated, with four receiving TC (two FDA, one DDA, one NC) and 56 undergoing PC (two FDA, 40 DDA, 14 NC), with 75% experiencing a reduction or cessation of drop attacks (Table 3).

Among 11 heterotopia patients, five had TC (0 FDA, three DDA, two NC) and five had PC (two FDA, one DDA, two NC), showing similar treatment rates and positive outcomes in 60% of cases. One patient with heterotopia underwent PC followed by TC and achieved freedom from drop attacks (one FDA) (Table 4). All 16 lissencephaly patients underwent TC, all of whom experienced a full remission of drop attacks (16 FDA) (Table 5). A single polymicrogyria patient also received TC, resulting in a complete remission of drop attacks (one FDA) (Table 6).

## Review

Discussion

The data indicate that PC is the most frequently employed surgical intervention for BMCD, utilized in 53% of the cases. TC appears to be particularly effective for certain BMCD subtypes, such as lissencephaly and polymicrogyria, where all patients experienced full remission of drop attacks. For tuberous sclerosis, most patients undergoing TC reported fewer drop attacks, with a significant portion achieving either reduction or cessation of symptoms. In contrast, PC was more commonly performed and demonstrated a high rate of positive outcomes in cortical dysplasia, with 75% of patients experiencing a reduction or cessation of drop attacks. This suggests that PC may be the preferred surgical approach for this BMCD subtype. Heterotopia patients showed mixed outcomes with both TC and PC, but a notable case involved a patient achieving freedom from drop attacks after undergoing both procedures sequentially.

Total Callosotomy

A retrospective study conducted in Beijing analyzed surgical treatment approaches to refractory epilepsy in patients with a diagnosis of Tuberous sclerosis for at least one year between 2001-2011. To minimize selection bias, all patients in the study had failed two types of antiepileptic drugs for six months. The age of surgical patients was between five and 28 years, which may have reduced confounding via large age differences in the study participants [2]. Total callosotomy, along with tuber resection, was performed in 11 cases, and all patients were followed post-operatively at the one-year and five-year mark. Patients who underwent TC between 2001 and 2006 also received a 10-year follow-up. The lack of a 10-year follow-up in the cases between 2007 and 2011 raises information bias concerns. The study was one of the first to show that surgical treatment showed better seizure control than medical treatment at the end of the five-year follow-up [2]. This study focuses on varying treatment modalities for epilepsy in tuberous sclerosis patients. Because callosotomy was only performed as an adjunctive treatment with tuber resection in these cases, it is not reflected in the data analysis but is presented to demonstrate the efficacy that the procedure may have in these patients.

Iwasaki et al. conducted a study with a patient population age range of one year and five months to 24 years, which found that earlier performance of total callosotomy in patients with infantile or early childhood-onset epilepsy had a greater incidence of complete curability [7]. All patients in this study were diagnosed with West syndrome. Likewise, Sato et al. described a three-year-old with diagnosed tuberous sclerosis who was refractory to anti-epileptic drugs (AEDs) and underwent TC and tuber resection. Post-operative imaging revealed further tubers that were later resected as well. After TC and tuber resection, the patient was successfully seizure-free on AEDs. This study highlights the importance of thorough pre-operative imaging and EEG analysis before surgery, as TC combined with tuber resection may be most effective in treating epilepsy associated with malformations of cortical development [8]. The small population size of these studies increases the risk of selection bias.

A study conducted by Kawai et al. on patients with drop attacks associated with MCDs showed a similar pattern of intractable epilepsy with medical management [1]. Following TC, patients had complete remission of drop attacks. The follow-up period for this study was between 1.4 and 5.8 years. This difference in follow-up raises concern for information bias.

Taken together, these four papers highlight the importance of further studies to be conducted on the long-term remission of refractory epilepsy in patients with MCD following TC. A case report of two patients with MCDs and associated refractory epilepsy who underwent laser interstitial thermal therapy and corpus callosotomy, compared to the standard craniotomy followed by callosotomy, is presented [9]. They report a successful demonstration of this less invasive surgical approach compared to callosotomy. Further longer-term monitoring and larger sample sizes are important in substantiating this case report. A study reported three children who underwent complete radiosurgical callosotomy between 2000 and 2004. All three children had MCDs, with one having Lennox-Gastaut syndrome and two having hemispheric cortical dysplasia. The median age of onset of epilepsy in these children was 5.5 months. All three children suffered significant mental developmental delay, and gamma knife callosotomy was performed. The study states that two of the three children had significant improvement within a 15-month post-operative period. The small sample size and short reported post-operative follow-up period raise concerns for possible information bias in this retrospective study [10].

Several papers have found indications for total callosotomy in the treatment of refractory epilepsy secondary to MCDs. One paper describes endoscopic-assisted interhemispheric transcallosal hemispherectomy as a major advancement in callosotomy. It provides evidence in a growing area of study focusing on the benefit of endoscopy guidance in epileptic surgery for treating hemimegalencephaly [3]. In a follow-up paper, Chandra et al. describe the benefit of endoscopy-guided epileptic surgery via complete callosotomy in patients with lissencephaly [11].

Patients with MCDs that are bilateral/diffuse, which include lissencephaly, polymicrogyria, and subcortical band heterotopia, are at greater risk of developing medically refractory epilepsy [12]. A study enrolling 23 patients divided them into five groups based on their initial presentations and diagnoses pre-operatively. Most of the participants had severe mental deficits from their MCDs. Group 5, which included patients with subcortical band heterotopia and drop attacks, showed a beneficial response to total callosotomy in most of the patients. This study also found that pre-operative imaging modalities are important in understanding patient seizure type [12]. Another case study by Baba et al. focused on total callosotomy in patients suffering from diffuse bilateral polymicrogyria. The benefit of this study was that it followed the three patients for several years up until the time of publication. Therefore, a clearer long-term outlook was described following total callosotomy. All three patients showed improvement in functionality following total callosotomy [13].

Partial Callosotomy

A retrospective study conducted in Bangkok found partial callosotomy proved effective in treating patients with epilepsy. The study included both patients with partial and generalized seizures before surgical treatment [14]. The inclusion of patients with different seizure types raises questions regarding the importance of treating different seizure types as confounding variables and performing appropriate measures, including randomization or matching, to ensure that this variable is accounted for when analyzing the effect of study outcomes. Furthermore, Lin and Kwan reviewed partial callosotomy procedures between July 1993 and November 1996. Patient seizure history pre- and post-callosotomy was gathered from patients’ families. This study emphasized the complex function of the corpus callosum in the development of epilepsy in patients suffering from Lennox-Gastaut syndrome [15]. The complexity of the corpus callosum was further emphasized by a case report that showed PC to improve symptoms of epilepsy in a patient with congenital bilateral perisylvian cortical dysplasia [16].

Subcortical laminar heterotopia is a form of bilateral MCD due to abnormal neuronal migration [17]. The first reported study of partial callosotomy to treat refractory epilepsy in this patient population was published in 1993 [17]. Another study compared partial callosotomy to lamotrigine medical therapy. Of the two patients who underwent partial callosotomy, only one had complete remission of both atonic and tonic-clonic seizures. They report that one patient placed on lamotrigine therapy without callosotomy also had remission from tonic-clonic seizures and status epilepticus. This study, published in 1999, is important in understanding why callosotomy treatment has recently been focused on treating atonic seizure types refractory to medical management [18]. Lastly, Bernasconi et al. conducted a case series review published in 2001 and reported no significant improvement in epilepsy following partial callosotomy [19]. This provided further research on partial callosotomies in larger populations.

*Both (Partial and Total Callosotomy* *)*

Pearsson et al. conducted a retrospective study comparing seven callosotomy patients, five of whom underwent total callosotomy. They reported four of the seven patients as completely free from drop attacks following callosotomy. Two of the patients who underwent callosotomy were not included in the study as one passed away by the time of the study and another did not provide informed consent for inclusion in the study. Therefore, this study did not spend much time comparing partial versus total callosotomy but does highlight the efficacy of the surgical intervention in treating refractory epilepsy [20]. Another retrospective study was conducted wherein pediatric patients underwent callosotomy between 1983 and 2002 to treat drop attacks and severe generalized convulsive seizures. This study reports that 91% of total callosotomy patients and 67% of partial callosotomy patients were completely free of drop attacks post-operatively. Total callosotomy was superior in this review, and no adverse neurologic effects were seen following TC as opposed to PC [21]. Lastly, Matsuhashi et al. reported a patient with subcortical band heterotopia with refractory epilepsy that further emphasizes the efficacy of total callosotomy over partial callosotomy. This patient first underwent partial callosotomy, which did not improve her epilepsy. She was then referred for total callosotomy, and six months post-operatively, she no longer had any atonic seizures. Despite success in treating her epilepsy, callosotomy did not seem to improve her severely low developmental status [22].

There were several limitations of this systematic review, such as only selecting studies published in English and the inability to control for all demographic variables. Information regarding patient sex, comorbidities, specific cortical pathology, and anti-seizure medication protocol was collected to minimize the effect of confounding variables. However, further studies are warranted to explore the effect of total versus partial callosotomy in patients of different sexes, ages, and pharmacologic protocols. Additionally, future studies should focus on elucidating the optimal timing, extent, and technique of corpus callosotomy, while also exploring adjunctive therapies and novel approaches to enhance seizure control and improve the quality of life for this challenging patient population. By addressing these knowledge gaps, we can advance the field of epilepsy surgery and ultimately provide better outcomes for individuals living with BMCD-related epilepsy. Nevertheless, our findings reveal a diverse landscape of surgical approaches and outcomes, highlighting the complexities of optimizing seizure control while minimizing adverse neuropsychological effects.

## Conclusions

This systematic review of the literature on corpus callosotomy for atonic drop seizures in BMCD provides valuable insight into the surgical management of medically refractory epilepsy. PC emerges as a promising intervention, particularly in cases of severe refractory epilepsy associated with bilateral MCDs. Studies supporting PC demonstrate significant improvements in seizure cessation, with some patients achieving long-term remission. However, total callosotomy also presents as a viable option, showing effectiveness in certain cases of epilepsy associated with BMCD. While not as extensively studied as PC, TC exhibits favorable outcomes in selected patients, suggesting its potential utility in tailored treatment strategies.

Overall, our review underscores the importance of individualized surgical decision-making guided by a thorough understanding of the underlying pathology, seizure phenotype, and neurocognitive profile of patients with BMCD.
